# Improved Comprehensive Performance of GaN-Based E-Mode HEMTs with a Thin Al_2_O_3_ Interlayer

**DOI:** 10.3390/nano16161027

**Published:** 2026-08-19

**Authors:** Guang Qiao, Huaize Liu, Cheng Feng, Yufeng Liao, Ruiling Gong, Hui Guo, Pengfei Shao, Dunjun Chen

**Affiliations:** 1 The Key Laboratory of Third Generation Semiconductors and High Energy Efficiency Devices, Nanjing University, Nanjing 210093, China; 200700084@szut.edu.cn (G.Q.); liu_huaize@163.com (H.L.); ruiling_gong@smail.nju.edu.cn (R.G.); huiguo@nju.edu.cn (H.G.); djchen@nju.edu.cn (D.C.); 2 School of Electronic Science and Engineering, Nanjing University, Nanjing 210093, China; 3 School of Electronic and Information Engineering, Suzhou University of Technology, Changshu 215500, China

**Keywords:** AlGaN/GaN HEMT, Al_2_O_3_ interlayer, atomic layer deposition, dynamic on-resistance degradation, current collapse

## Abstract

In this paper, a 2.5 nm thick Al_2_O_3_ interlayer deposited by atomic layer deposition was inserted between AlGaN/GaN HEMT structure and SiN_x_ passivation layer to reduce interface damage of the semiconductor/dielectric introduced directly by plasma-enhanced chemical vapor deposition. It is found that this ultra-thin Al_2_O_3_ interlayer can not only obviously increase the output current and extrinsic transconductance by reducing the access-region resistance, but also effectively suppress current collapse and the threshold voltage drift due to fewer interface defects in the access region between the gate and drain. More importantly, dynamic on-resistance degradation of devices with an Al_2_O_3_ interlayer is significantly improved in comparison with the only Si_3_N_4_-passivated HEMTs without an Al_2_O_3_ interlayer.

## 1. Introduction

AlGaN/GaN high-electron mobility transistors (HEMTs) have been widely considered as potential candidates for the next generation of power electronics due to their fast switch speed, high conversion efficiency, high power density and high-voltage capabilities [1,2]. However, GaN-based HEMTs are still suffering some serious issues such as dynamic on-resistance (*R*_ON_) degradation and current collapse, which are critical in power switching system design and operation [3,4,5,6,7]. The degradation of the performance of these devices is closely related to passivation dielectrics. Among the various passivation dielectrics, PECVD SiN_x_ or SiO_2_ is commonly used as the mainstream passivation layer for GaN-based HEMTs in the industry [8,9,10,11,12]. However, plasma bombardment during the PECVD deposition process can damage the AlGaN surface and introduce additional interface states. Therefore, some high-k dielectrics (e.g., Al_2_O_3_, HfO_2_) deposited by atomic layer deposition (ALD) with less damage and thick SiN_x_/Al_2_O_3_ [13,14] bilayer dielectrics are often applied for GaN-based MOSFET devices as gate dielectrics to improve the semiconductor/dielectric interface, enhance gate control capability, and reduce gate leakage current. Their impacts on device performance have also been extensively studied [13,14,15,16,17,18]. However, the thickness of these dielectric layers does not exceed 30 nm, which is primarily attributed to the inherently low growth rate of ALD processes. Such a thin passivation layer is unfavorable for device reliability. The thick SiN_x_/Al_2_O_3_ [19] bilayer dielectrics are also used to reduce surface leakage current and trapping effects in GaN-based E-mode HEMT. However, for p-GaN gate E-mode HEMTs, the effects of ultra-thin Al_2_O_3_ passivation dielectrics in the access region on device performance, especially on dynamic *R*_ON_ degradation and current collapse, are rarely studied.

In this paper, a 2.5 nm thick Al_2_O_3_ interlayer deposited by ALD was inserted between the AlGaN/GaN HEMT structure and the common SiN_x_ passivation layer to reduce interface damage to the semiconductor/dielectric introduced directly by the SiN_x_ PECVD process. The comparison results show that this ultra-thin Al_2_O_3_ interlayer can not only significantly increase the output current, but also effectively suppress current collapse and dynamic *R*_ON_ degradation. The physical mechanisms for these improvements on the performance and reliability of GaN-based HEMTs induced by the ultra-thin Al_2_O_3_ interlayer are also analyzed by means of various electrical characterizations.

## 2. Experiment

**Figure 1:**
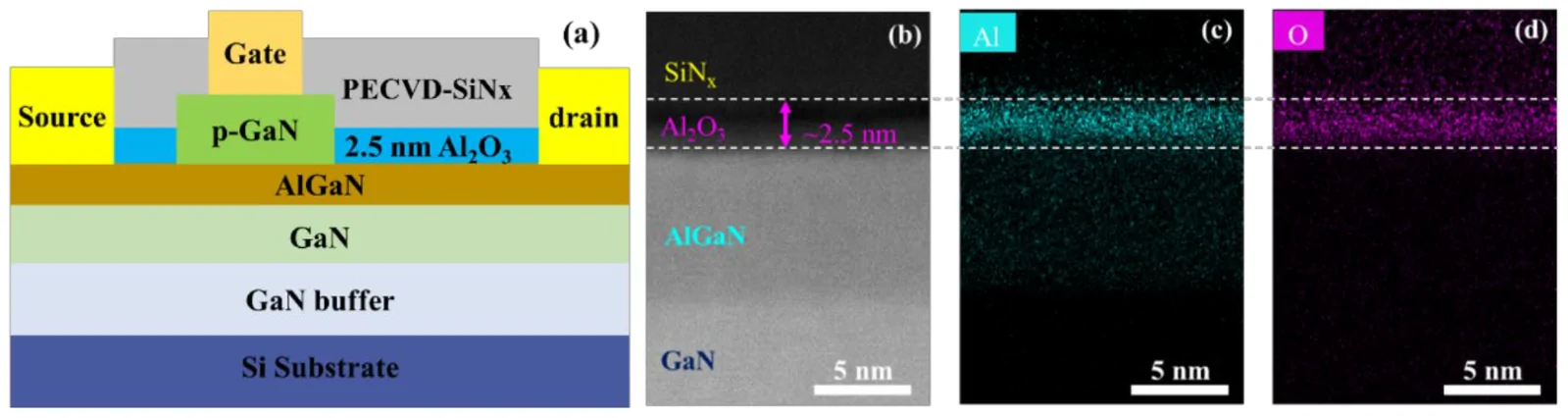
(**a**) Structure schematic diagram of GaN HEMTs with a Al_2_O_3_ interlayer, and TEM image (**b**), EDX mapping image of Al atom (**c**) and EDX mapping image of O atom (**d**).

The used AlGaN/GaN HEMT structure was grown by MOCVD on a 6-inch Si wafer with a 5.2 μm C-doping GaN buffer, a 300 nm UID-GaN channel, a 15 nm Al_0.25_Ga_0.75_N barrier, and a 70 nm p-GaN layer with Mg doping concentration of 1 × 10^19^ cm^−3^. A schematic diagram of the structure of AlGaN/GaN HEMTs is shown in Figure 1a. The fabrication process begins with an etch of the p-GaN layer outside the gate region by inductively coupled plasma. After a dilute HCl treatment, a thin Al_2_O_3_ dielectric layer is then deposited by ALD. Subsequently, ohmic contacts were formed by electron-beam evaporation of the Ti/Al/Ni/Au (20/120/50/100 nm) metal stack, followed by rapid thermal annealing at 850 °C for 30 s in N_2_ ambient, and the gate electrode is formed by evaporating a Ni/Au (20/100 nm) stack. Before metal electrodes were deposited, the Al_2_O_3_ dielectric layer in the electrode region was removed by extending the development time during the source/drain photolithography step. A 100 nm thick SiN_x_ passivation layer was deposited by PECVD. Mesa isolation was then achieved by inductively coupled plasma (ICP), and contact windows were subsequently opened by reactive ion etching (RIE). The fabricated GaN-based HEMTs are of L_GS_/L_G_/L_GD_ = 4/4/12 μm, respectively. To demonstrate the presence of the Al_2_O_3_ interlayer, cross-sectional transmission electron microscope (TEM) and the energy-dispersive X-ray (EDX) mapping of Al and O atoms of the Al_2_O_3_ interlayer were carried out in the fabricated GaN-based HEMTs with the Al_2_O_3_ interlayer, and the results are shown in Figure 1b–d, where a 2.5 nm thick Al_2_O_3_ layer can be clearly observed. Electrical characterizations were recorded by the Keithley 4200 semiconductor analyzer.

## 3. Results and Discussion

**Figure 2:**
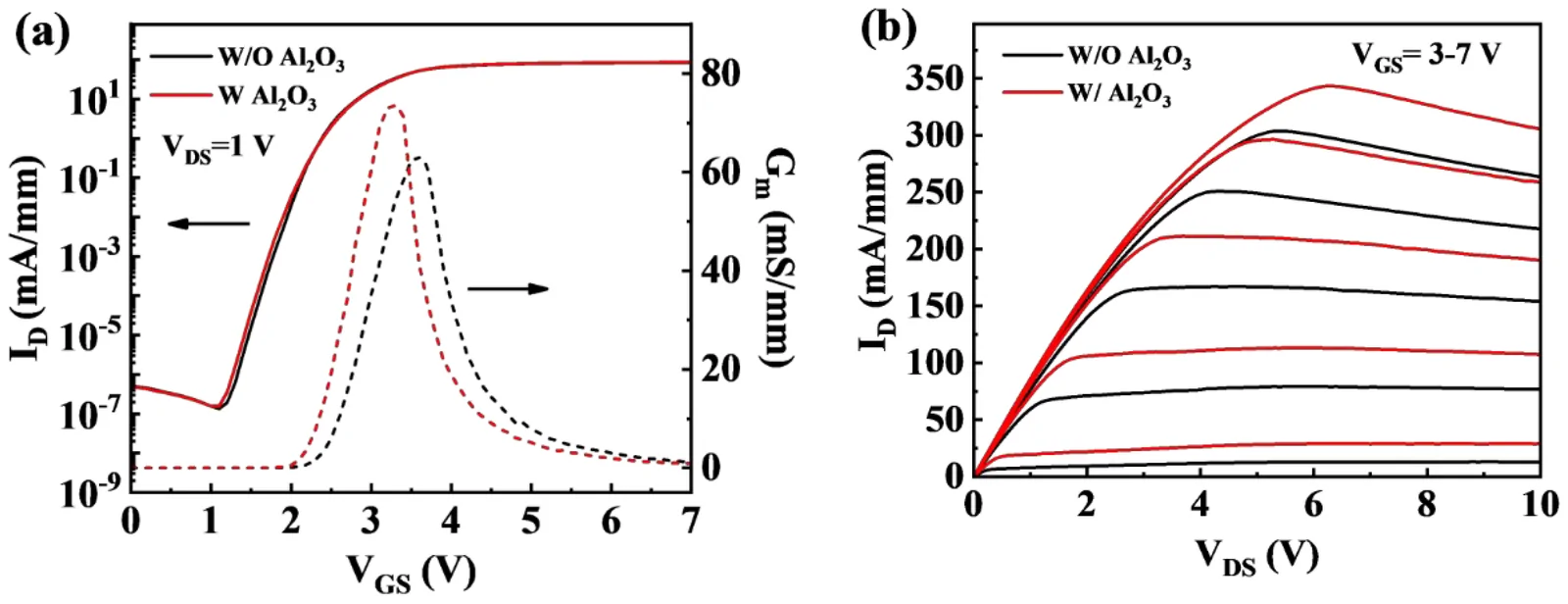
The comparative results of transfer (**a**) and output (**b**) characteristics of GaN-based HEMTs with and without Al_2_O_3_ interlayer.

The transfer and output characteristics of GaN-based HEMTs with and without a 2.5 nm Al_2_O_3_ inserting layer are shown in Figure 2a and Figure 2b, respectively. As shown in Figure 2a, both HEMTs exhibit almost the same positive threshold voltage (Vth) of about 2.1 V defined at I_D_ = 0.1 mA/mm, showing an enhancement-mode (E-mode) operation. The device with the Al_2_O_3_ interlayer achieves a higher extrinsic transconductance than its counterpart. Further, the device with the Al_2_O_3_ interlayer exhibits a maximum output current of 342 mA/mm at V_GS_ = 7 V that is approximately 13.2% higher than that of the device without the Al_2_O_3_ layer, along with a lower on-resistance, as shown in Figure 2b.

To clarify the origin of the higher extrinsic transconductance and output current, we performed the transfer length method (TLM) and variable L_GD_ resistance measurements. The normalized contact resistance extracted from the TLM measurement is R_C_ = 0.626 Ω⋅mm. The variable L_GD_ measurements were performed at V_GS_ = 7 V, and the extracted access-region sheet resistances R_sh_ are 681.37 and 844.32 Ω/□ for the devices with and without the Al_2_O_3_ interlayer, respectively. For the device geometry of L_GS_/L_G_/L_GD_ = 4/4/12 μm, the corresponding source series resistances (R_S_ = R_C_ + R_sh_L_GS_) are 3.35 and 4.00 Ω⋅mm. The total source and drain access resistances (R_access_ = R_sh_(L_GS_ + L_GD_)) are 10.90 and 13.51 Ω⋅mm, respectively. Thus, introducing the Al_2_O_3_ interlayer reduces the access-region resistance by approximately 19.3%. The resistance components are analyzed according to R_ON_ = 2R_C_ + R_access_ + R_CH_. These results indicate that the higher extrinsic transconductance and output current of the Al_2_O_3_ interlayer device are primarily associated with its lower access resistance.

**Figure 3:**
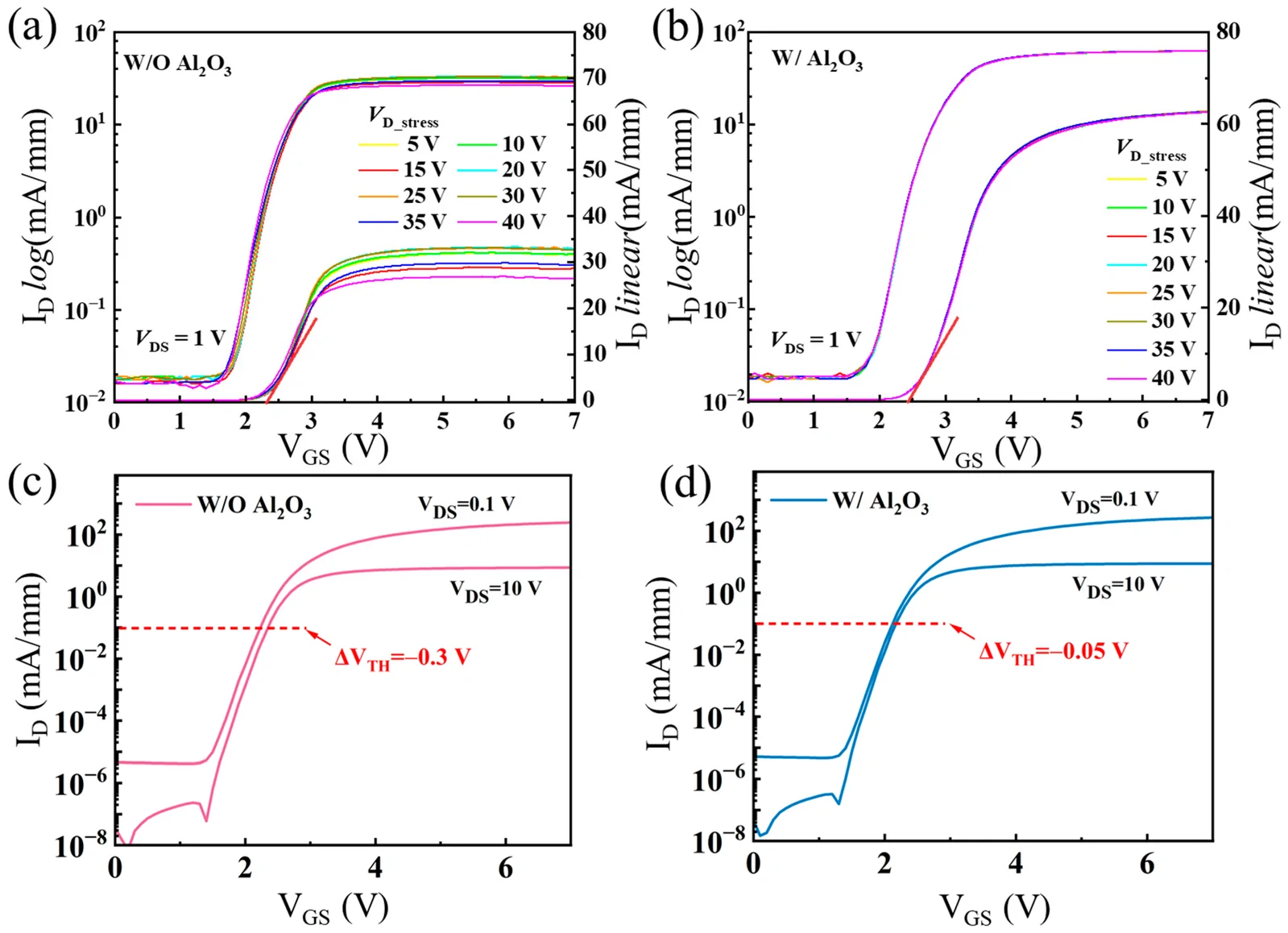
Threshold voltage characteristics of AlGaN/GaN HEMTs without (**a**) and with (**b**) the Al_2_O_3_ interlayer after different off-state drain-stress voltages (V_DS_,stress = 5–40 V) measured at a fixed V_DS_ = 1 V. Threshold voltage shift of the devices without (**c**) and with (**d**) the Al_2_O_3_ interlayer under different V_DS_.

Figure 3a,b show the transfer characteristics after off-state drain stress at V_GS_ = 0 V; with V_DS_, stress increased from 5 to 40 V. The transfer characteristics were measured at a fixed V_DS_ = 1 V. The device without the Al_2_O_3_ interlayer exhibits a V_TH_ shift of −0.12 ± 0.02 V, whereas no resolvable shift (0.00 ± 0.02 V) is observed for the device with the interlayer, indicating improved threshold voltage stability. Figure 3c,d compare the threshold voltage stability of the devices without and with the Al_2_O_3_ interlayer measured at V_DS_ = 0.1 and 10 V. As V_DS_ increases, the device without Al_2_O_3_ exhibits a negative V_TH_ shift of approximately 0.30 V, whereas the device with the Al_2_O_3_ interlayer shows a much smaller negative V_TH_ shift of approximately 0.05 V. These apparent shifts may include contributions from drain-induced barrier lowering (DIBL), series resistance, and changes in the operating regime. This behavior may be associated with a weaker influence of the drain electric field on the channel beneath the gate thanks to the improved interface quality between the semiconductor and dielectric through inserting a thin Al_2_O_3_ passivation layer [20].

**Figure 4:**
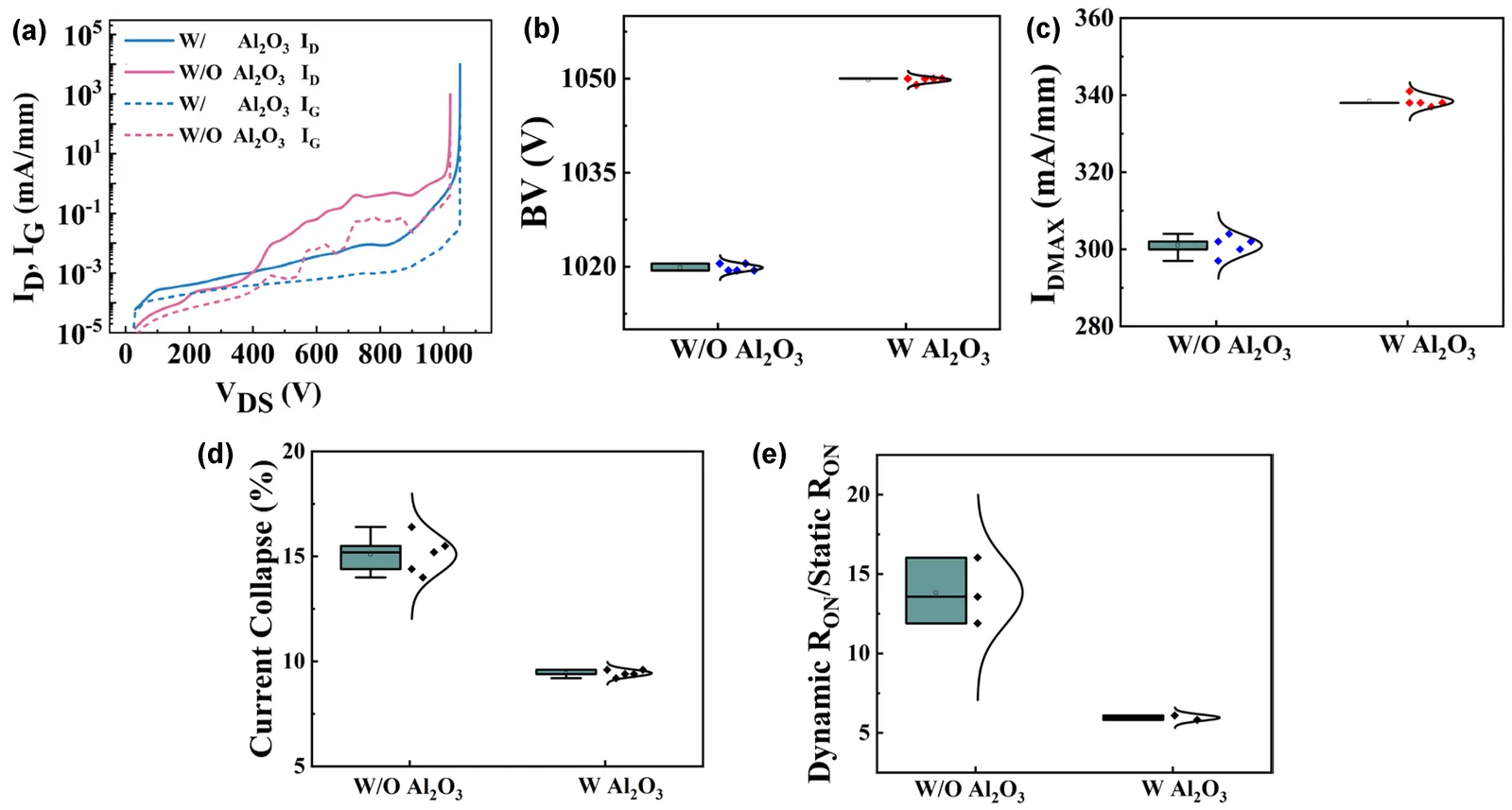
(**a**) The off-state breakdown characteristics of the devices (L_GD_ = 12 μm) with and without the Al_2_O_3_ interlayer measured with the substrate grounded. The measurements were conducted at room temperature with V_GS_ = 0 V and the substrate grounded. The breakdown voltage is defined at the hard-breakdown point where the off-state leakage current exhibits a sharp increase to 10 mA/mm; (**b**) the error bars of breakdown voltage; (**c**) the error bars of maximum output current at V_GS_ = 7 V; (**d**) the error bars of current collapse; (**e**) the error bars of dynamic R_ON_/static R_ON_.

Figure 4a shows the off-state breakdown characteristics of the devices (L_GD_ = 12 μm) with and without the 2.5 nm Al_2_O_3_ interlayer. Both devices exhibit hard-breakdown characteristic at the voltage points of 1050 V and 1020 V with substrate grounded, respectively, almost no difference in breakdown voltage. But there is an obvious difference in their off-state leakage current characteristics, where the device with the Al_2_O_3_ interlayer features a lower leakage current increase with increasing drain voltage. In the low-drain-to-source voltage range, the device with the Al_2_O_3_ interlayer presents a higher leakage current; a possible reason is that fewer electrons are captured by the interface traps due to fewer interface defects in the access region after inserting the thin Al_2_O_3_ layer. Therefore, the device with the Al_2_O_3_ interlayer exhibits a higher leakage current off-state in the low-drain-to-source voltage range. However, under higher drain voltage, the dielectric/semiconductor interface lateral leakage by the electron hopping conduction mechanism begins to play a more important role and results in higher leakage current in the device without the Al_2_O_3_ interlayer. It should be noted that the tested device parameters were taken from the average level of multiple devices in this work, and typically, we plotted the error bars of breakdown voltage and maximum output current at V_GS_ = 7 V of the two devices with and without the Al_2_O_3_ interlayer, as shown in Figure 4b and Figure 4c, respectively. The error bars of current collapse and the dynamic-to-static R_ON_ ratio are shown in Figure 4d and Figure 4e, respectively. However, the values of current collapse and the dynamic-to-static R_ON_ ratio are more discrete due to the consistency of the devices and the complexity of testing.

**Figure 5:**
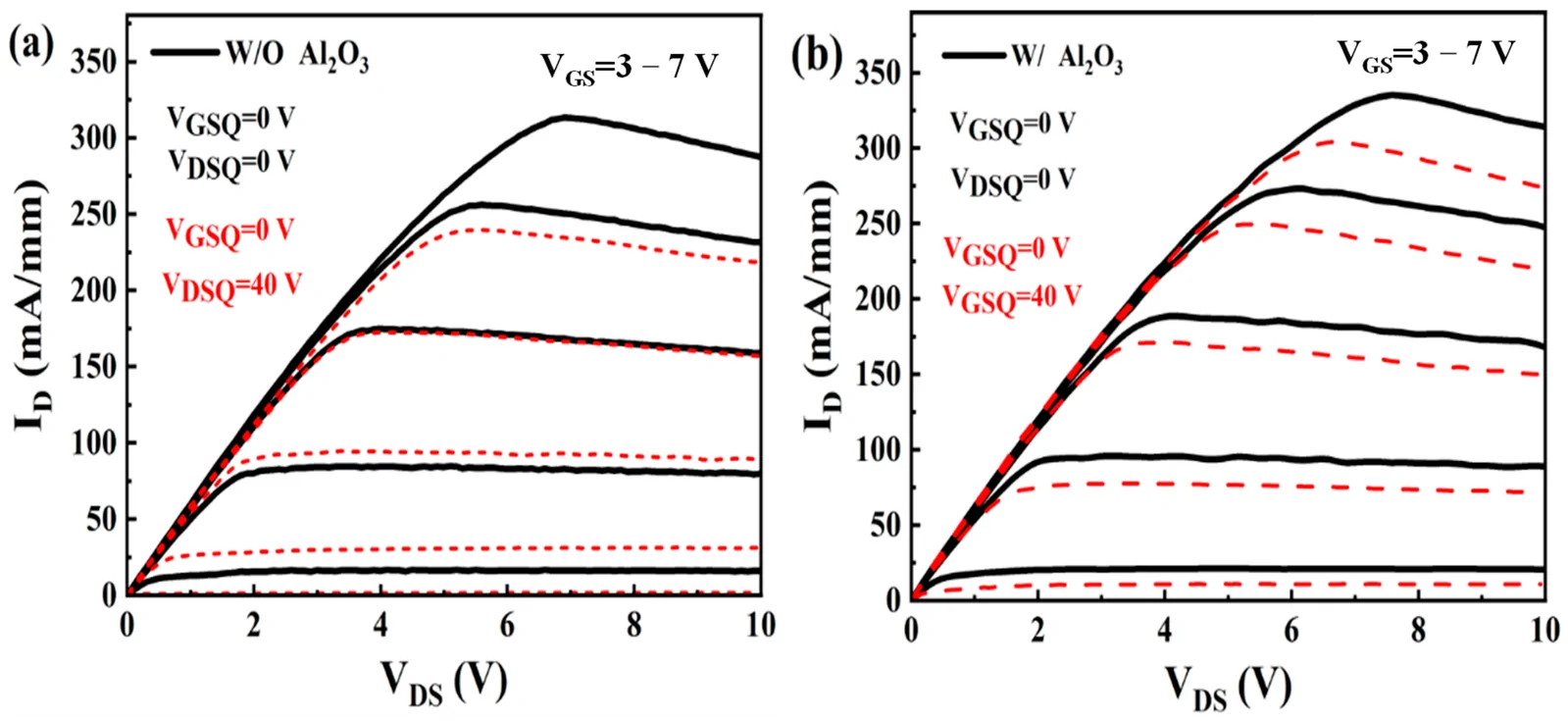
Pulsed I-V characteristics of the devices with and without the Al_2_O_3_ interlayer, measured at two quiescent bias points: (**a**) V_GSQ_/V_DSQ_ = 0 V/0 V and (**b**) V_GSQ_/V_DSQ_ = 0 V/40 V.

The current collapse phenomenon is usually observed in GaN-based HEMTs and results in reliability degradation [21,22]. Figure 5a,b show the pulsed I-V characteristics of the devices with and without the Al_2_O_3_ interlayer measured from two quiescent bias points, V_GSQ_/V_DSQ_ = 0 V/0 V and 0 V/40 V, respectively. The measurements were performed in the pulsed mode using a pulse width of 100 μs and a pulse period of 1 ms, corresponding to a duty cycle of approximately 10%. During the on-state measurement, V_GS_ was varied from 3 to 7 V in 1 V step, while V_DS_ was swept from 0 to 10 V. Under the off-state quiescent bias, both devices exhibit a reduction in drain current, indicating off-state stress-induced current collapse. At V_GS_ = 7 V, the drain current of the device without Al_2_O_3_ decreases from 313 to 239 mA/mm, corresponding to a current collapse of approximately 24%, as shown in Figure 5a. In contrast, the device with the Al_2_O_3_ interlayer shows a much smaller decrease from 335 to 306 mA/mm, corresponding to a current collapse of approximately 9%, as shown in Figure 5b. The substantially reduced current collapse in the device with the Al_2_O_3_ interlayer suggests that the interlayer effectively mitigates off-state-stress-induced charge trapping. The ultra-thin Al_2_O_3_ layer can passivate the AlGaN surface and may also protect it from plasma-induced damage during the subsequent PECVD-SiN_x_ deposition. By preventing direct contact between PECVD-SiN_x_ and AlGaN, the interlayer may reduce surface/interface-related defects and electron trapping under off-state high-field stress in the gate-to-drain access region, thereby alleviating depletion of the underlying 2DEG and suppressing current collapse [23,24].

**Figure 6:**
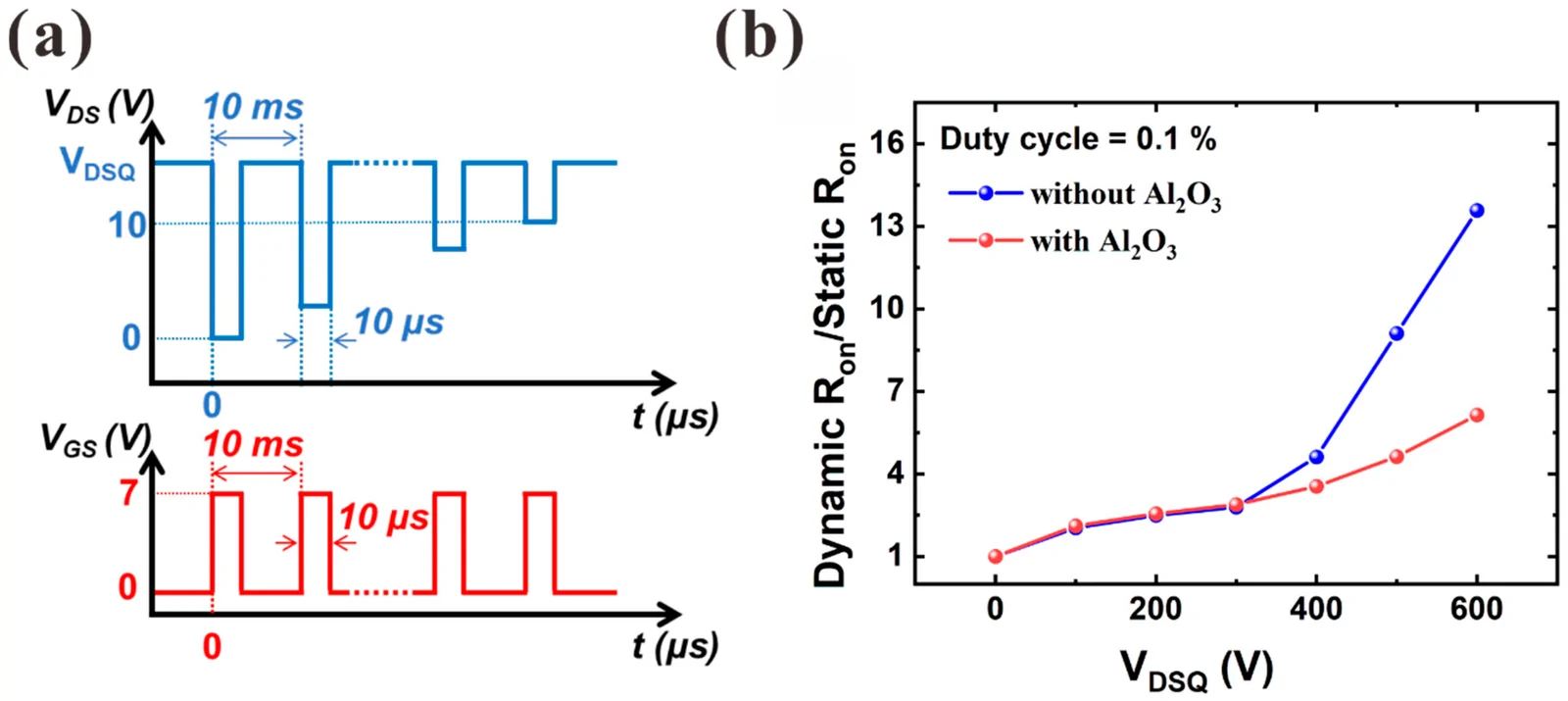
(**a**) Pulsed I-V waveforms of *V*_GS_ and *V*_DS_; (**b**) dynamic *R*_ON_/static *R*_ON_ of the devices with and without the Al_2_O_3_ interlayer.

Dynamic *R*_ON_ degradation is currently the biggest obstacle for GaN power devices to move towards large-scale applications [25,26]. It is well known that traps in the C-doping GaN buffer and at the dielectric/AlGaN interface are the primary causes of dynamic *R*_ON_ degradation [27]. However, in this work, the buffer layer for the two devices with and without the Al_2_O_3_ interlayer is completely identical, fabricated on the same wafer. Therefore, the effect from the buffer layer on dynamic *R*_ON_ degradation is the same and will no longer be analyzed separately here. The dynamic performance of HEMT devices with and without the Al_2_O_3_ interlayer is characterized using an AMCAD AM3200 series pulsed I-V system. The devices are switched between the off-state and on-state with a pulse width of 10 μs and a period of 10 ms, as shown in Figure 6a. In comparison with the device without the Al_2_O_3_ interlayer, the HEMT with the Al_2_O_3_ interlayer exhibits much better dynamic *R*_ON_ degradation. Under *V*_DS_ stress below 300 V, the on-resistance degradation of the two devices shows no significant difference, keeping a dynamic-to-static *R*_ON_ ratio below 2.5. However, after *V*_DS_ stress exceeding 300 V, the device without the Al_2_O_3_ interlayer undergoes severe dynamic *R*_ON_ degradation, and in contrast, the device with the Al_2_O_3_ interlayer exhibits a better dynamic stability with a dynamic-to-static *R*_ON_ ratio below six at a *V*_DS_ of up to 600 V, demonstrating that the thin Al_2_O_3_ interlayer deposited by ALD can reduce interface damage and effectively passivate the AlGaN surface states, and hence substantially improve the dynamic *R*_ON_ degradation of the GaN-based HEMTs.

## 4. Conclusions

In this work, the effects of a 2.5 nm thick Al_2_O_3_ passivation interlayer on the performance of GaN-based HEMTs have been investigated through dynamic and static electrical testing. The measurement results indicate that the ultra-thin Al_2_O_3_ passivation layer can not only obviously increase the output current and extrinsic transconductance by reducing the access-region resistance, but also effectively suppress current collapse and dynamic *R*_ON_ degradation. These improvements on the performance and reliability of the GaN-based HEMTs can be generally attributed to the reduced interface damage and uniform and dense passivation effect to the AlGaN surface states by the ALD technique.

## Data Availability

The original contributions presented in this study are included in the article. Further inquiries can be directed to the corresponding author.
